# Effects of Delta-Like Noncanonical Notch Ligand 1 Expression of Human Fetal Liver Hepatoblasts on Hematopoietic Progenitors

**DOI:** 10.1155/2019/7916275

**Published:** 2019-03-18

**Authors:** Jörg C. Gerlach, Robert L. Thompson, Bruno Gridelli, Eva Schmelzer

**Affiliations:** ^1^Department of Surgery, University of Pittsburgh, Pittsburgh, Pennsylvania 15203, USA; ^2^Department of Bioengineering, University of Pittsburgh, Pittsburgh, Pennsylvania 15203, USA; ^3^University of Pittsburgh Medical Center (UPMC), University of Pittsburgh, Pittsburgh, 15213 Pennsylvania, USA; ^4^Istituto di Ricovero e Cura a Carattere Scientifico-Istituto Mediterraneo per i Trapianti e Terapie ad Alta Specializzazione (IRCCS-ISMETT), UPMC Italy, 90127 Palermo, Italy

## Abstract

Although the hepatic and hematopoietic progenitors of the liver are well characterized, the interactions between these two lineages remain mostly elusive. Hepatoblasts express delta-like noncanonical Notch ligand 1 (Dlk1), whose cleaved extracellular domain can become a soluble protein. We assessed the effects of DLK1 gene expression knockdown in cultures of total fetal liver cells. Furthermore, we separated Dlk1^+^ hepatoblasts from the total liver cell fraction and investigated effects of direct cell contact. Dlk1^−^ cells were cultured either without Dlk1^+^ hepatoblasts, in direct contact with hepatoblasts, or separated from hepatoblasts by a porous membrane in inserts to inhibit cell contact but allow free exchange of molecules. Expression of the hepatic and hematopoietic genes, colony forming unit potential of various hematopoietic progenitors, and cell numbers and types were investigated. We found that DLK1 knockdown in total fetal liver cell cultures decreased total cell numbers. The expression of hepatic progenitor genes and mature hematopoietic genes was affected. Hematopoietic BFU-E and CFU-GM colony numbers were reduced significantly. The depletion of Dlk1^+^ hepatoblasts in culture decreased the potential of all hematopoietic progenitors to form colonies of all types and reduced the percentage of mature hematopoietic cells. The addition of hepatoblasts in inserts to Dlk1^−^ cells further decreased the potential to form the CFU-GM and CFU-GEMM colonies and the percentage of mature hematopoietic cells but increased total cell numbers. Conclusively, direct contact of Dlk1 supports hematopoietic progenitor expansion and functionality that cannot be reconstituted in coculture without direct cell contact.

## 1. Introduction

During fetal liver development, hepatic stem cells give rise to transient hepatic progenitors, hepatoblasts [1, 2]. Whereas hepatic stem cells are negative for the delta-like noncanonical Notch ligand 1 (Dlk1), fetal hepatoblasts are strongly Dlk1-positive [3]. Postnatally, hepatoblasts become mature hepatocytes, which are completely Dlk1-negative. Dlk1, also known as preadipocyte factor 1, is a transmembrane surface molecule containing multiple epidermal growth factor repeats [4]. The extracellular domain can be cleaved by ADAM17 (disintegrin and metalloproteinase domain-containing protein 17) or TACE (tumor necrosis factor-*α*-converting enzyme), giving rise to a soluble fragment. In adipogenesis, this soluble fragment has been shown to inhibit the differentiation of preadipocytes into adipocytes, while the membrane-bound Dlk1, however, represses preadipocyte proliferation [5, 6].

The fetal liver is the primary organ for erythropoiesis during development. Concurrent with the progression of hepatic parenchymal maturation, the liver ceases hematopoiesis and the bone marrow becomes the main site of hematopoiesis in the adult. In early embryonic development, Dlk1 is expressed in the smooth muscle layer of the dorsal aorta and the ventral subaortic mesenchyme of the aorta-gonad-mesonephros region; Mirshekar-Syahkal et al. showed that in this region, Dlk1 negatively affected the hematopoietic stem and progenitor cells [7]. These effects required the membrane-bound form of Dlk1, but the soluble Dlk1 had no impact. The peaking and ceasing of hematopoiesis in the fetal liver appear simultaneously with the peaking and ceasing of Dlk1 expression of hepatoblasts, strongly suggesting a tight interaction between both lineages. Chou et al. demonstrated that mouse E15.5 Dlk1-positive sorted cells supported the expansion of CD150^+^CD48^−^CD41^−^ hematopoietic progenitors from the bone marrow [8] and fetal liver [9]. Although short-term expansion could be recapitulated by a conditioned medium, the physical contact between the two cell types was required for long-term expansion of CD150^+^CD48^−^CD41^−^ hematopoietic progenitors.

Here, we investigated the impact of Dlk1 expression of hepatoblasts on different hematopoietic progenitor cell types of the human fetal liver. Our data indicate that Dlk1^+^ hepatoblasts play a significant role in the regulation of hematopoietic stem cell proliferation and differentiation, which is dependent on direct cell contact.

## 2. Materials and Methods

### 2.1. Immunohistochemistry

To confirm Dlk1 expression in hepatoblasts *in vivo*, we costained human fetal liver sections for a hepatoblast-specific marker alpha-fetoprotein (AFP) with Dlk1. Unfixed liver tissue was embedded in an OCT compound (Sakura Finetek, Torrance, CA) and flash frozen in liquid nitrogen-cooled isopentane followed by freezing in liquid nitrogen. Sections of 5 to 10 *μ*m thickness were cut, air-dried, fixed in ice-cold acetone/methanol (1 : 1 (*v*/*v*)) at -20°C for 10 min, and then air-dried. Sections were blocked with 10% goat serum (Thermo Fisher Scientific, Waltham, MA) and a 10% FcR block (Miltenyi Biotec, Bergisch Gladbach, Germany) in phosphate-buffered saline (PBS). Primary antibodies used were rabbit IgG anti-human AFP (Thermo Fisher Scientific) and mouse IgG2b anti-human Dlk1 (R&D Systems, Minneapolis, MN), which were applied in a blocking buffer. Washing steps between stainings included three washes with PBS 0.1% Triton X-100. Secondary antibodies included goat anti-rabbit AF546 and anti-mouse IgG2b AF488 (Thermo Fisher Scientific) and were applied in the blocking buffer including 4′,6-diamidino-2-phenylindole (DAPI) (Sigma-Aldrich, St. Louis, MO) as a nuclear stain. Sections incubated with isotype-matched immunoglobulins served as negative controls. Sections were embedded in Aquamount (Polysciences, Warrington, PA) and coverslipped. Images were acquired by confocal microscopy using an Olympus Fluoview 1000 system and software version 3.0.2.0 (Olympus, Center Valley, PA).

### 2.2. Liver Cell Isolation

The general procedure for isolation of human fetal liver cells has been described previously [1]. We made slight modifications to the procedure, essentially as described in the methods of Gerlach et al. [10]. The human fetal livers of 17 to 20 weeks of gestational age were obtained as anatomical gifts provided by the Allegheny Reproductive Health Center, Pittsburgh, PA. The organs were retrieved from abortions after informed consent from donors and approval by the local Institutional Review Board. Liver tissue was scraped with scalpels and digested to single cells in a digestion medium containing 0.6 mg/mL collagenase type IV, 120 U/mL DNase, 1% fatty acid-free bovine serum albumin (BSA), 30 nM selenium (Sigma-Aldrich), 100 units/mL penicillin, 100 *μ*g/mL streptomycin, and 0.25 *μ*g/mL amphotericin B in a RPMI 1640 medium (Life Technologies, Carlsbad, CA) at 37°C. Cell suspensions were washed two times with the RPMI 1640 medium (containing 1% fatty acid-free BSA, 30 nM selenium, 100 units/mL penicillin, 100 *μ*g/mL streptomycin, and 0.25 *μ*g/mL amphotericin B) by centrifugation at 300g and subsequently filtered through 40 *μ*m pore size cell strainers (Becton Dickinson, Bedford, MA).

### 2.3. Cell Numbers and Viability

Cell numbers and viabilities were determined using a Neubauer chamber and trypan blue (Life Technologies) exclusion in phase-contrast microscopy (Zeiss Invertoskop C, Carl Zeiss, Jena, Germany).

### 2.4. DLK1 Knockdown

To investigate the effects of expression of DLK1, its gene expression was knocked down using specifically targeting siRNA in cell culture. Total fetal liver cells were plated in six-well plastic culture plates (Corning, Lowell, MA) coated with 5 *μ*g/cm^2^ rat tail collagen-1 (Becton Dickinson) at a density of 50,000 cells/cm^2^. The cultures included either 1 *μ*M DLK1-targeting siRNA (Dharmacon Accell SMARTpool containing a mix of four differently gene-specific targeting sequences provided by the manufacturer (GE Healthcare Life Sciences, Lafayette, CO)), 1 *μ*M negative control scrambled nontargeting siRNA (Dharmacon Accell SMARTpool containing a mix of four nontargeting sequences provided by the manufacturer), or no siRNA negative control. siRNA was delivered in Accell Delivery Medium including antibiotic-antimycotic (100 units/mL penicillin, 100 *μ*g/mL streptomycin, and 0.25 *μ*g/mL amphotericin B). Cells were cultured for three and five days. Subsequently, cells were analyzed for gene expression, numbers, viability, and marker expression using flow cytometry. In addition, cells were subjected to colony forming unit assays.

### 2.5. Magnetic-Activated Cell Sorting

Dlk1-positive hepatoblasts were isolated from total fetal liver cell suspensions using magnetic-activated cell sorting (MACS). Freshly isolated total liver cell suspensions were washed and resuspended in a washing buffer (PBS without Ca and Mg, 2 mM EDTA, and 0.5% BSA, pH 7.2 (Sigma-Aldrich)). Suspensions were incubated for 15 min at 4°C in the washing buffer with an FcR block (Miltenyi Biotec) to prevent an unspecific receptor-mediated antibody binding, DNase to prevent cell aggregation, and a mouse IgG1 anti-Dlk1 antibody (Thermo Fisher Scientific). After washing, cells were incubated for 15 min at 4°C with anti-mouse IgG magnetic beads (Miltenyi Biotec). Cells were washed and separated on LS columns using a Midi magnet (Miltenyi Biotec) according to the manufacturer's protocols. The effluent contained cells negative for Dlk1, and retained cells on the column were Dlk1-positive hepatoblasts. Fractions were analyzed for purity using flow cytometry.

### 2.6. Cell Culture

For experiments investigating the effects of direct contact between Dlk1^+^ hepatoblasts and Dlk1-negative cells, magnetically separated Dlk1^+^ hepatoblasts and Dlk1^−^ cells were cultured in three different settings. Dlk1^−^ cells were placed into the bottom wells of Transwell permeable supports (Corning). We applied 10% Dlk1-positive hepatoblasts directly to the Dlk1^−^ cells either into the bottom wells or the top inserts; negative controls did not receive cells into the inserts. Inserts had a pore size of 0.4 *μ*m, allowing for the free exchange of molecules but preventing cell contact. Culture plates were coated with collagen-1 as described for siRNA experiments. Cells were cultured for five days in the supplemented RPMI 1640 medium containing 5% fetal bovine serum, 0.1% fatty acid-free BSA, 30 nM selenium, 540 *μ*g/mL niacinamide, 5 ng/mL insulin, 10 ng/mL transferrin, free fatty acid mixture (2.36 *μ*M palmitic acid, 0.21 *μ*M palmitoleic acid, 0.88 *μ*M stearic acid, 1.02 *μ*M oleic acid, 2.71 *μ*M linoleic acid, and 0.43 *μ*M linolenic acid), 0.1 *μ*M hydrocortisone, 50 *μ*M *β*-mercaptoethanol (Sigma-Aldrich), 2 mM Glutamax, 100 units/mL penicillin, 100 *μ*g/mL streptomycin, and 0.25 *μ*g/mL amphotericin B (Life Technologies).

### 2.7. Gene Expression Analyses

Cells were lysed directly with a RLT buffer including 1% beta-mercaptoethanol (Sigma-Aldrich), and nucleic acids were isolated using the shredder and isolation columns (AllPrep DNA/RNA Mini Kit, Qiagen, Valencia, CA), including DNA digestion by DNase treatment on columns. Concentrations of nucleic acids were determined fluorometrically using the Quant-iT Assay kits and Qubit fluorometer (Thermo Fisher Scientific). RNA was reverse transcribed to cDNA with High-Capacity cDNA Reverse Transcription Kit (Applied Biosystems, Carlsbad, CA). Gene expression was analyzed with real-time PCR using the StepOnePlus system and software version 2.0 and a predesigned TaqMan probe and primer assay mixes with a gene expression master mix (Applied Biosystems). Beta-actin served as a housekeeping gene for internal normalization. TaqMan assay mixes used were ACTB (beta-actin), AFP (alpha-fetoprotein), CCNE1 (cyclin E1), CD34 (cluster of differentiation 34), DLK1 (delta-like noncanonical Notch ligand 1), EPCAM (epithelial cell adhesion molecule; CD326), GYPA (glycophorin A; CD235a), KRT19 (keratin 19, type 1; cytokeratin 19), MKI67 (marker of proliferation Ki-67), PECAM1 (platelet and endothelial cell adhesion molecule 1; CD31), PTPRC (protein tyrosine phosphatase, receptor type C; CD45), and VWF (von Willebrand factor). Expression was quantified using the ddCt method. Freshly isolated total human fetal liver cells served as positive controls; no template (water) was used as a negative control. Each of the biological samples was analyzed with two technical repeats. Data are presented as knockdown compared to controls without knockdown (100%) for each respective gene.

### 2.8. Colony Forming Unit Assay

Colony forming unit (CFU) assays were performed to investigate the potential of different hematopoietic progenitors to form colonies of various hematopoietic lineages. Cells were cultured in ultralow attachment dishes (STEMCELL Technologies, Vancouver, Canada). A complete MethoCult methylcellulose-based assay was used according to the manufacturer's instructions (STEMCELL Technologies). For the investigation of effects of DLK1 knockdown, cells were collected after five days of incubation with siRNA and added to methylcellulose. For the investigation of the effects of physical contact of Dlk1^+^ cells on hematopoietic cells (as described above), Dlk1^−^ cells were collected after five days of culture and added to methylcellulose. After 14 days of culture, colonies were classified and counted on gridded dishes using phase microscopy (Zeiss Invertoskop C, Carl Zeiss, Jena, Germany). Four different types of colonies were identified, including the CFU-E (colony forming unit-erythrocyte), BFU-E (burst forming unit-erythrocyte), CFU-GM (colony forming unit-granulocyte and macrophage), and CFU-GEMM (colony forming unit-granulocyte, erythrocyte, macrophage, and megakaryocyte) colonies.

### 2.9. Flow Cytometry

Cells were analyzed using flow cytometry to determine the percentages of specific hepatic and hematopoietic cell types. Cells were suspended in Brilliant Stain Buffer (Becton Dickinson) that included a 10% human FcR-blocking reagent (Miltenyi Biotec) and antibodies or respective isotype controls. Cells were incubated for 15 min at 4°C in the dark, washed, and resuspended in a buffer for analysis (0.5% BSA (Sigma-Aldrich), 2 mM disodium EDTA (Sigma-Aldrich) in PBS without calcium and magnesium (Life Technologies)). Antibodies used were mouse IgG1 and 2b lineage cocktail (Lin)-FITC, mouse IgG1 CD34-PE, mouse IgG1 CD38-AF700, mouse IgG1 CD31-BV421, mouse IgG1 CD45-BUV395 (all Becton Dickinson), mouse IgG1 CD326-PE (Miltenyi Biotec), and rabbit IgG Dlk1-AF647 (Bioss Antibodies, Woburn, MA); isotypes were mouse IgG1&2b-FITC, IgG1-PE, IgG1-AF700, IgG1-BV421, IgG1-BUV395 (all Becton Dickinson), and rabbit IgG-AF647 (Bioss Antibodies). A FACSAria II flow cytometer (Becton Dickinson) and FlowJo software version 10.4.1 (Tree Star, Ashland, OR) were used to analyze cells. An initial forward versus side scatter gate was applied to exclude cell debris and doublets. Compensation beads (Becton Dickinson) were used to compensate for fluorochrome spectral overlap. Negative controls included nonstained cells and cells incubated with isotype controls.

### 2.10. Statistical Analyses

Data are given as means from *n* biological repeats ± standard deviation. Student's *t*-test was used to analyze statistically significant differences. ^∗^, ^∗∗^, and ^∗∗∗^ indicate statistically significant differences (*p* ≤ 0.05, *p* ≤ 0.01, and *p* ≤ 0.001, respectively).

## 3. Results

On average, from one human fetal liver tissue donation of gestational weeks 17–20, we obtained 1.99 × 10^9^ ± 0.20 × 10^9^ total cells with a viability of 97%±1% (*n* = 7).

**Figure 1:**
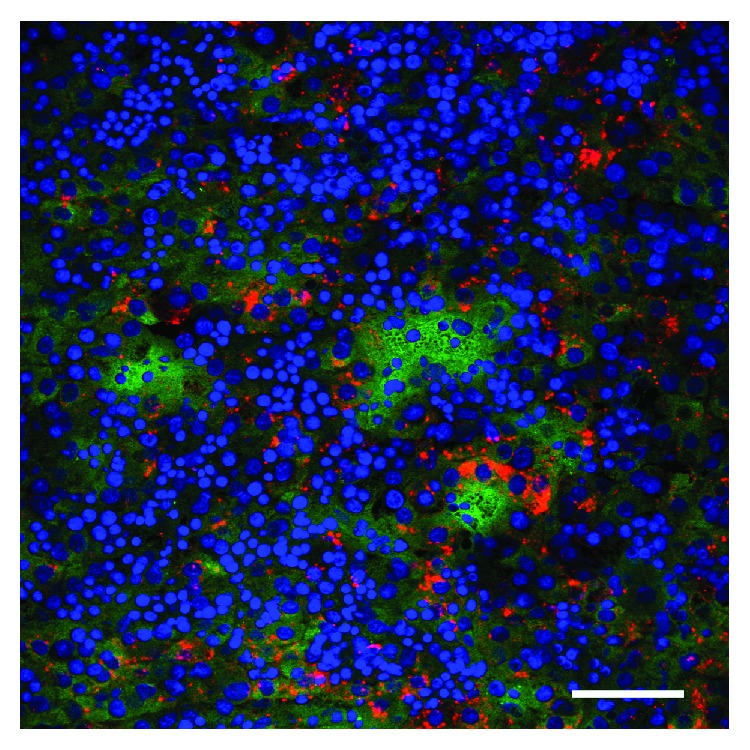
Expression of Dlk1 in the human fetal liver. Hepatoblasts of human fetal liver sections were stained for Dlk1 (green) and alpha-fetoprotein (red); cell nuclei were stained with DAPI (blue). Confocal fluorescence microscopy, scale bar: 50 *μ*m.

We validated Dlk1 expression in human fetal liver tissue (Figure 1). Parenchymal hepatoblasts that were positive for AFP also coexpressed Dlk1.

### 3.1. Effects of Knockdown of DLK1 in Total Fetal Liver Cell Cultures

**Figure 2:**
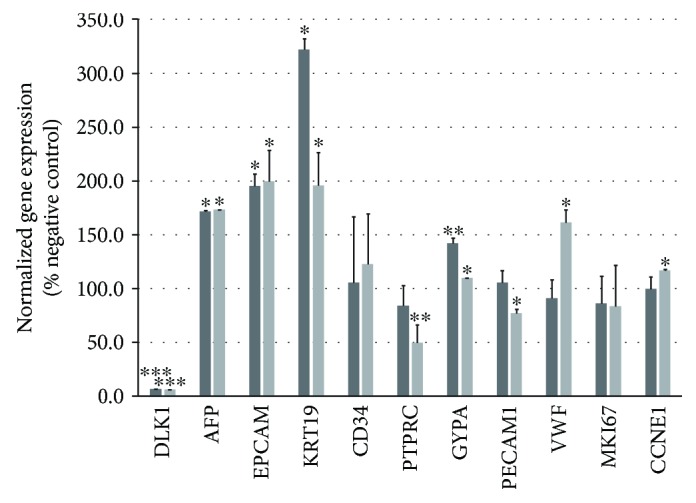
Gene expression of human fetal liver cells after DLK1 knockdown. Total human fetal liver cells were cultured for three (dark grey bars) or five (light grey bars) days with DLK1-targeting siRNA and analyzed for their expression of the hepatic, hematopoietic, endothelial, and cell cycle genes. Gene expression is given as percent of the respective control (100%) that received nontargeting control siRNA. Data are given as means from *n* = 3 different repeats ± standard deviation. ^∗^, ^∗∗^, and ^∗∗∗^ indicate statistically significant differences (*p* ≤ 0.05, *p* ≤ 0.01, and *p* ≤ 0.001, respectively). Abbreviations: AFP: alpha-fetoprotein; CCNE1: cyclin E1; CD34: cluster of differentiation 34; DLK1: delta-like noncanonical Notch ligand 1; EPCAM: epithelial cell adhesion molecule, CD326; GYPA: glycophorin A, CD235a; KRT19: keratin 19, type 1, cytokeratin 19; MKI67: marker of proliferation Ki-67; PECAM1: platelet and endothelial cell adhesion molecule 1, CD31; PTPRC: protein tyrosine phosphatase, receptor type C, CD45; VWF: von Willebrand factor.

When total fetal liver cells were cultured with DLK1-targeting siRNA, the expression of the various hepatic and hematopoietic genes was affected (Figure 2). The siRNA-mediated knockdown reduced DLK1 gene expression to 6-7% of controls after three and five days in culture. DLK1 knockdown affected the expression of several analyzed genes. After three and five days in culture, the hepatic progenitor genes AFP, EPCAM, and KRT19 were increased significantly. The mature hematopoietic gene PTPRC was decreased significantly, but GYPA, typically expressed by erythrocytes, was increased; CD34 expression was not affected. After five days in culture, PECAM1, which is expressed by hemangioblasts, endothelial progenitors, and endothelial cells, was slightly reduced; VWF, which is expressed only by endothelial cells, was increased. The cell proliferation marker MKI67 was not affected by knockdown, and the expression of the cell cycle progression marker CCNE1 was slightly increased after five days in culture.

**Figure 3:**
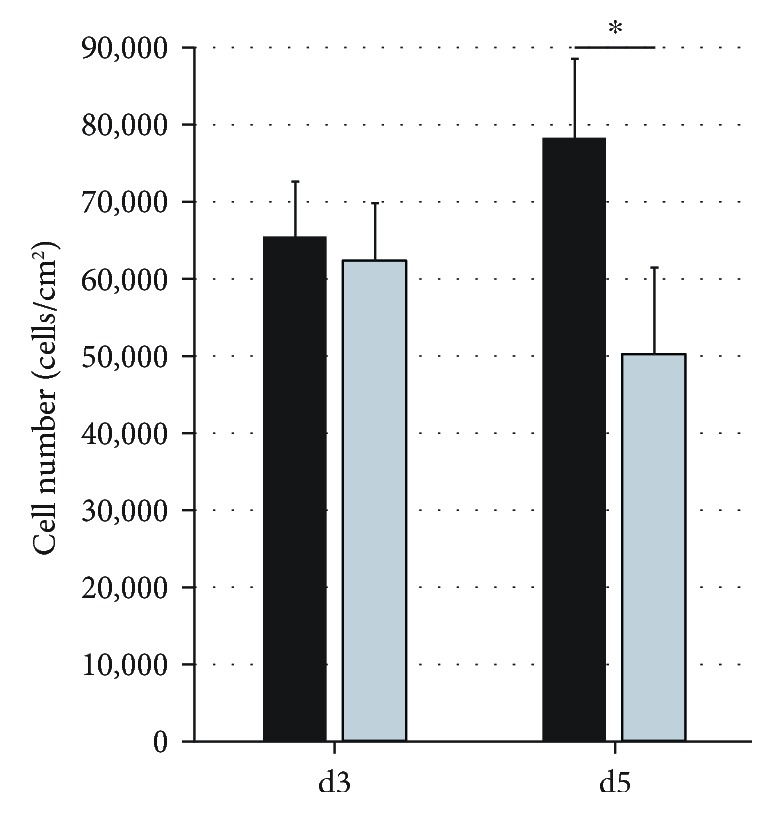
Total cell numbers of human fetal liver cells after DLK1 knockdown. Total human fetal liver cells were cultured for three and five days with DLK1-targeting siRNA (light grey bars) or nontargeting control siRNA (black bars), and total cell numbers were determined. Data are given as means from *n* = 3 biological repeats ± standard deviation. ^∗^ indicates a statistically significant difference (*p* ≤ 0.05).

We further investigated the effects of knockdown on total cell numbers. While we observed in controls an increase in cell numbers, DLK1 knockdown significantly reduced the total overall cell numbers after five days in culture (Figure 3) without affecting cell viability, which was at least 95.4% for all experiments.

**Figure 4:**
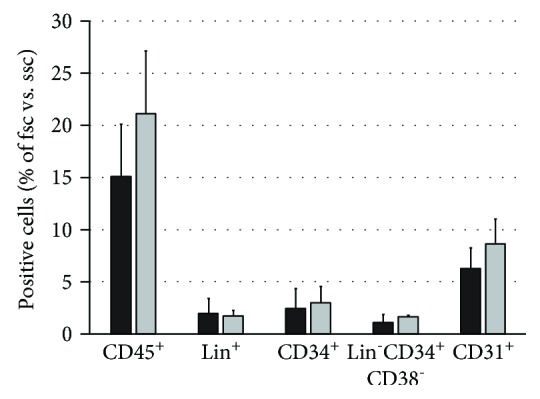
Flow cytometry analysis of human fetal liver cell cultures after DLK1 knockdown. Total human fetal liver cells cultured with DLK1-targeting siRNA (grey bars) or nontargeting control siRNA (black bars). Cells were analyzed for expression of hematopoietic CD45, lineage (Lin) surface antigens, CD34, CD31, and Lin^−^CD34^+^CD38^−^ (hematopoietic stem cells). Data are given as means ± standard deviation from *n* = 3 biological repeats. Abbreviations: fsc: forward scatter; ssc: side scatter; CD: cluster of differentiation.

When cell types were investigated using flow cytometry (Figure 4), we could not find significant effects on the percentages of hematopoietic cell types, including the CD45^+^, Lin^+^, CD34^+^, CD31^+^, and Lin^−^CD34^+^CD38^−^ hematopoietic stem cells, suggesting that those cell types were about equally reduced in their numbers.

**Figure 5:**
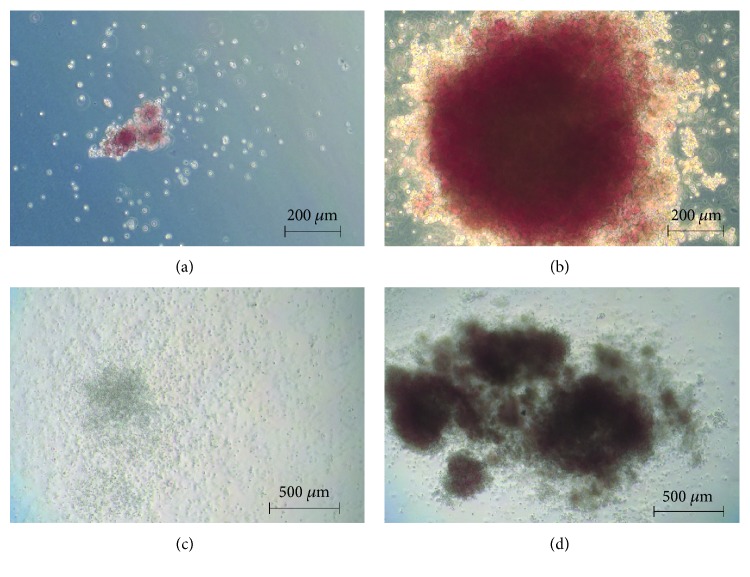
Representative examples of colony types in CFU assays. Human fetal liver cells were subjected to colony forming unit (CFU) assays that included a complete set of hematopoietic growth factors in a methylcellulose medium. Four different types of colonies could be identified and counted in light microscopy. (a) Small CFU-E colony that derives from a more mature erythroid progenitor; (b) large BFU-E colony that originates from an immature erythrocyte progenitor; (c) CFU-GM colony that originates from a primitive granulopoietic progenitor and does not contain erythrocytes; (d) CFU-GEMM colony that derives from the most primitive hematopoietic progenitor, forming very large colonies containing all four lineages.

DLK1 knockdown, however, affected the CFU potential of the hematopoietic fraction. Hematopoietic progenitors from the fetal liver can give rise to distinct hematopoietic lineages when subjected to a 14-day culture of CFU assays that include a complete set of hematopoietic growth factors in a methylcellulose medium. In general, four different types of colonies can be identified and enumerated (Figure 5): small (up to 200 cells) CFU-E colonies that derive from a more mature erythroid progenitor; large (more than 200 cells) BFU-E colonies that originate from an immature erythrocyte progenitor; CFU-GM colonies that originate from a primitive granulopoietic progenitor and do not contain erythrocytes; and CFU-GEMM colonies that derive from the most primitive hematopoietic progenitor, forming very large colonies containing all four lineages.

**Figure 6:**
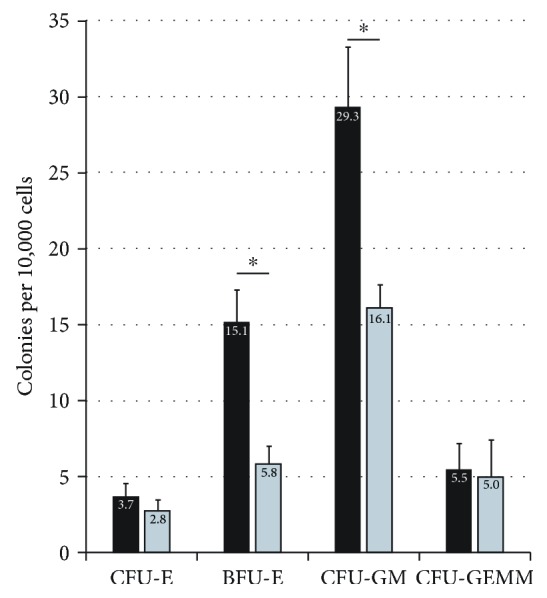
Colony forming unit assays after DLK1 knockdown. Total human fetal liver cells were cultured for five days with DLK1-targeting siRNA (grey bars) or nontargeting control siRNA (black bars). CFU assays were carried out, and hematopoietic colonies were identified in light microscopy. Data are given as means ± standard deviation from *n* = 3 biological repeats. ^∗^ indicates a statistically significant difference (*p* ≤ 0.05).

When cells were subjected to DLK1 knockdown (Figure 6), we could not detect significant effects on the colony formation capacity of CFU-Es and CFU-GEMMs, but we observed a significant reduction in BFU-E and CFU-GM colony numbers.

### 3.2. Effects of Separation of Dlk1^+^ Hepatoblasts from Hematopoietic Cells

To investigate the requirement for direct contact of membrane-bound Dlk1 of hepatoblasts to affect hematopoietic progenitors, we separated Dlk1-positive hepatoblasts from total human fetal liver cells using MACS. Purities of sorted fractions were confirmed using flow cytometry (Dlk1-positive: 94 ± 3%; Dlk1-negative: 95 ± 2%). We set up three different culture models that included the use of Transwell cell culture inserts. Dlk1-negative cells were plated in the bottom of the cell culture well. Dlk1-negative cells are mainly hematopoietic cell types but also include minor fractions of other fetal liver cells such as endothelial or stellate cell progenitors. Three different cultures were initiated: Dlk1-positive cells were added directly into the bottom of the well so that both fractions had direct contact with each other; Dlk1-positive cells were added into the upper insert, allowing for the free exchange of soluble molecules but preventing cell-cell contact; and no Dlk1-positive cells were added to the Dlk1-negative fraction. Cells were cultured for five days and analyzed for gene expression using real-time PCR, cell numbers and types were evaluated using flow cytometry, and hematopoietic colony formation potential was investigated.

**Figure 7:**
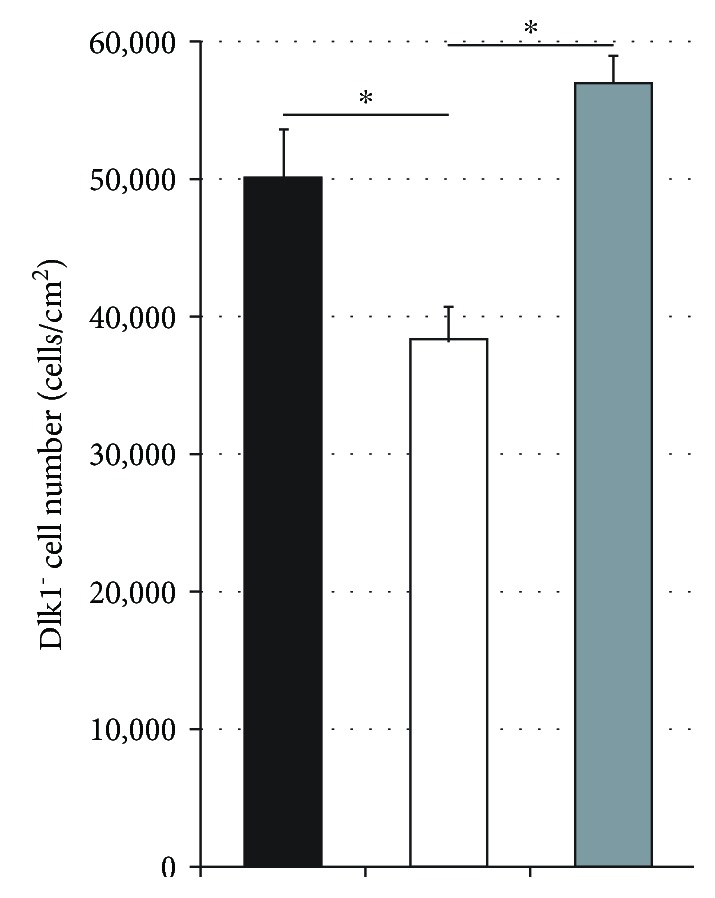
Cell numbers after Dlk1^+^ cell depletion. Numbers of Dlk1-negative human fetal liver-derived cells after five days in culture with Dlk1^+^ cells in direct contact (black bars), without Dlk1^+^ cells (white bars), or with Dlk1^+^ cells in inserts (grey bars). Data are given as means from *n* = 4 biological repeats ± standard deviation. ^∗^ indicates a statistically significant difference (*p* ≤ 0.05).

The depletion of Dlk1-positive hepatoblasts significantly reduced the number of Dlk1-negative cells compared to that of Dlk1-negative cells in direct contact with Dlk1-positive hepatoblasts (Figure 7). The addition of Dlk1-positive hepatoblasts to inserts led to a significant increase in numbers of Dlk1-negative cells compared to controls of Dlk1-negative cells without Dlk1-positive hepatoblasts in coculture.

**Figure 8:**
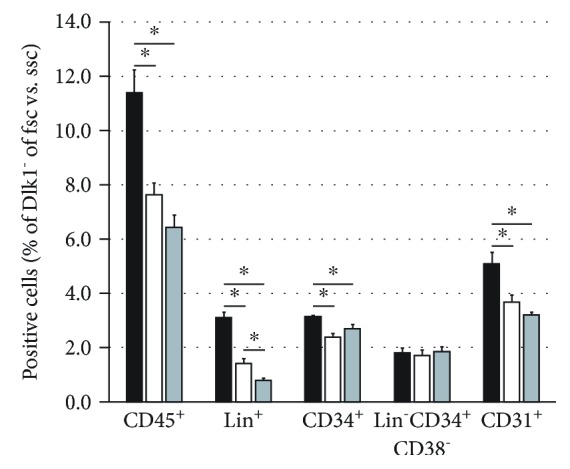
Flow cytometry analysis after Dlk1^+^ cell depletion. Flow cytometry analysis of Dlk1-negative human fetal liver-derived cells after five days in culture with Dlk1^+^ cells in direct contact (black bars), without Dlk1^+^ cells (white bars), or with Dlk1^+^ cells in inserts (grey bars). Data are given as means from *n* = 4 biological repeats ± standard deviation. ^∗^ indicates a statistically significant difference (*p* ≤ 0.05). Abbreviations: fsc: forward scatter; ssc: side scatter; CD: cluster of differentiation.

Next, we investigated the effects of the depletion of Dlk1-positive hepatoblasts on various hematopoietic cell types in flow cytometry (Figure 8). We analyzed the CD45^+^, Lin^+^, CD34^+^, CD31^+^, and Lin^−^CD34^+^CD38^−^ hematopoietic stem cells. The depletion led to a significant decrease in CD45^+^, Lin^+^, and CD31^+^ cells. Moreover, the addition of Dlk1-positive hepatoblasts to inserts did not increase the percentages of hematopoietic cells again but further decreased the percentages of lineage-positive hematopoietic cell types.

**Figure 9:**
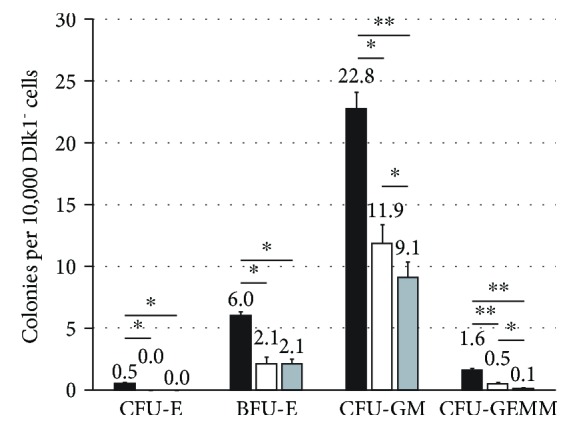
Colony forming unit assays after Dlk1^+^ cell depletion. CFU assays of Dlk1-negative human fetal liver-derived cells in five days preculture with Dlk1^+^ cells in direct contact (black bars), without Dlk1^+^ cells (white bars), or with Dlk1^+^ cells in inserts (grey bars). Data are given as means from *n* = 4 different repeats ± standard deviation. ^∗^ and ^∗∗^ indicate statistically significant differences (*p* ≤ 0.05 and ^∗∗^*p* ≤ 0.01, respectively). Abbreviations: CFU-E: colony forming unit-erythrocyte; BFU-E: burst forming unit-erythrocyte; CFU-GM: colony forming unit-granulocyte and macrophage; CFU-GEMM: colony forming unit-granulocyte, erythrocyte, macrophage, and megakaryocyte.

Furthermore, we also found that the depletion of Dlk1-positive hepatoblasts reduced the potential of the Dlk1-negative hematopoietic fraction to form colonies (Figure 9). Interestingly, the addition of hepatoblasts into inserts neither improved nor retained the hematopoietic potential but further decreased the potential to form the CFU-GM and CFU-GEMM colonies.

In conclusion, these data demonstrate that Dlk1 expressed by hepatoblasts affects specific hematopoietic progenitor cell types of the human fetal liver and that, potentially, soluble Dlk1 rather than membrane-bound Dlk1 has adverse effects on the hematopoietic fraction. Knockdown of DLK1 gene expression in total fetal liver cell cultures increased the expression of typical hepatic progenitor genes and decreased expression of hematopoietic PTPRC. Furthermore, it decreased overall cell numbers but affected no specific cell type of those that we analyzed. Also, it significantly reduced BFU-E and CFU-GM colony formation capacity. The physical separation of Dlk1-positive hepatoblasts from hematopoietic cells led to a decreased potential of the hematopoietic fraction to form colonies of all types. The addition of hepatoblasts in inserts to the Dlk1-negative cells increased the total number of Dlk1-negative cells, further reduced the percentage of mature hematopoietic lineage-positive cells, and resulted in a further decrease of the potential of hematopoietic stem cells to form the CFU-GM and CFU-GEMM colonies.

## 4. Discussion

Originally, Dlk1 was described in adipogenesis, where the extracellular soluble fragment inhibits the differentiation of preadipocytes into adipocytes and the membrane-bound Dlk1 inhibits preadipocyte proliferation [5, 6]. In the liver, Dlk1 is expressed transiently by fetal hepatoblasts [3], indicating a similar inductive role during liver development, as has been described for adipogenesis. Indeed, our findings indicate that direct contact of Dlk1 supported the expansion and functionality of hematopoietic progenitors; furthermore, this effect could not be recapitulated in coculture without direct cell contact.

Stromal cell lines have been investigated for their capacity to support the maintenance of hematopoietic stem cells [11–13]. Among several lines, only one was able to support the long-term maintenance of hematopoietic stem cells. This line was discovered to express Dlk1 [14]. Interestingly, it was demonstrated that the addition of the extracellular domain of Dlk1 as soluble protein had no effects on hematopoietic stem cells, and only when transfected cell lines expressed membrane-bound surface Dlk1 could they elicit effects on hematopoietic stem cells. Similarly, Brouard et al. [15] described a mouse liver-derived population positive for CD51, VCAM-1, E-cadherin, c-met, hepatocyte nuclear factor 4, albumin, alpha-fetoprotein, cytokeratin 18, and Dlk1, but negative for CD45, TER119, CD31, and PDGFR*α*. This expression profile characterizes hepatoblasts. The described cell fraction supported the production of megakaryocytes from mouse bone marrow hematopoietic stem cells and human peripheral blood. These findings corroborate our results that direct contact of hematopoietic stem cells with hepatoblasts, which are the primary fetal Dlk1-positive parenchymal cell type, is necessary to positively affect hematopoietic stem cells.

The mechanistic action that Dlk1 elicits on hematopoietic stem cells needs still to be discovered, and it is yet unclear if Dlk1 indeed causes direct effects on hematopoietic stem cells. As Chou et al. also suggest [8], it is also possible that hepatoblasts express additional surface molecules that could potentially affect hematopoietic stem cell maintenance. They considered Notch ligands and investigated the expression of all Notch ligands but did not find any of these to be enriched by Dlk1-positive cells. Furthermore, noticeable parallels of Dlk1 to the hepatic stem cell surface marker [1, 2] CD326 (or EPCAM (epithelial cell adhesion molecule)) seem to exist. Similar to Dlk1 expression in hepatoblasts, the extracellular domain of CD326 can be cleaved by ADAM17 [16, 17]. This causes the release of the short intracellular domain [18], which subsequently acts within the nucleus by inducing cell proliferation through upregulation of c-myc and cyclin A/E in nonhepatic tumorigenic and tumor cell lines [19]. We showed recently [10] that this mechanism does not induce proliferation in normal human fetal liver cells positive for CD326, in contrast to tumor cells; moreover, gene knockdown or treatment with an inhibitor of ADAM17 rather increased the number of CD326-positive normal fetal liver cells. To our knowledge, no data has been published on the effects of either the membrane-bound or soluble extracellular domain of Dlk1 on other liver cell types, as has been shown for CD326. Certainly, it is of further interest to investigate if the intracellular domain of Dlk1, after cleavage of its extracellular domain, is released and elicits effects similar to CD326.

## 5. Conclusions

We examined the effects of Dlk1 expression of hepatoblasts on hematopoietic progenitor cell types of the human fetal liver. Our data indicate that Dlk1^+^ hepatoblasts play an important role in the regulation of hematopoietic stem cell proliferation and differentiation, which was dependent on direct cell contact. The direct contact of Dlk1 supported hematopoietic progenitor expansion and functionality, which could not be reproduced in coculture without direct cell contact. These findings could also be relevant to the *ex vivo* expansion of hematopoietic progenitors for clinical applications.

## Data Availability

The data used to support the findings of this study are available from the corresponding author upon request.
